# Mixed-Phase Crystallization and Resonance Tuning in Sn-Incorporated GST Thin Films: Experimental Study with THz Metasurface Simulation

**DOI:** 10.1021/acsomega.6c01419

**Published:** 2026-06-01

**Authors:** Nantarat Srisuai, Kamonchanok Duangkanya, Yuttana Intaravanne, Chanunthorn Chananonnawathorn, Khwanchai Tantiwanichapan, Jirawat Assawakhajornsak, Hideki Nakajima, Mati Horprathum

**Affiliations:** † Spectroscopic and Sensing Devices Research Group, 54782 National Electronics and Computer Technology Center, Pathum Thani 12120, Thailand; ‡ 530102 Synchrotron Light Research Institute (Public Organization), Nakhon Ratchasima 30000, Thailand

## Abstract

Tin (Sn) was incorporated into Ge_2_Sb_2_Te_5_ (GST) thin films to create a tunable platform for terahertz (THz) sensing. The films were prepared by DC magnetron cosputtering and examined using energy-dispersive X-ray spectroscopy (EDS), X-ray diffraction (XRD), and X-ray photoelectron spectroscopy (XPS) to analyze composition and phase evolution. Results show that Sn modifies the crystallization pathway of GST, forming mixed SnTe and SnSb phases that enable phase transformation at a lower thermal energy. THz time-domain spectroscopy (THz-TDS) revealed a pronounced dielectric contrast between amorphous and crystalline states, with Sn incorporation enhancing phase transition. The measured optical parameters were implemented in simulations of split-ring resonator (SRR) metasurfaces containing a GST underlayer. Phase switching in pure GST produced a resonance shift of about 0.16 THz; however, the Sn-incorporated GST sample could improve modulation efficiency with reduced thermal input. These findings identify Sn-incorporated GST as a promising material for reconfigurable, energy-efficient THz device platforms.

## Graphical abstract

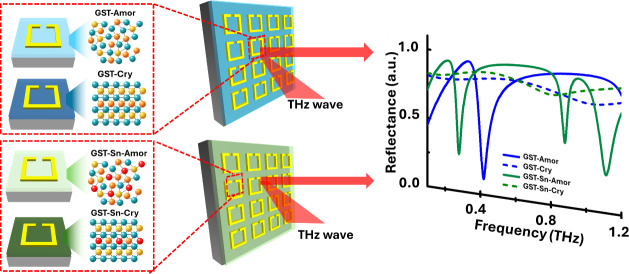

## 1 Introduction

The terahertz (THz) frequency range (0.1–10 THz) has become a focus of research because of its broad potential in areas such as noninvasive imaging, spectroscopy, and emerging wireless communication. One of the major challenges in advancing THz technology is the lack of materials and device platforms that can dynamically control electromagnetic waves in this spectral window.^1,2^ Phase-change materials (PCMs) have emerged as a strong candidate, as their reversible transition between amorphous and crystalline phases leads to pronounced changes in both optical and electrical properties.^3–5^

Among materials investigated for THz tunability, vanadium dioxide (VO_2_) has been frequently employed as a representative PCM due to its sharp and reversible metal–insulator transition near 68 °C, producing strong modulation of electrical and optical properties. However, the transition in VO_2_ is volatile, as the material reverts to its initial state once the external stimulus is removed, limiting its suitability for nonvolatile switching.^6–9^ Alongside VO_2_, graphene has also been investigated in THz tunable devices because of its outstanding conductivity and gate-tunable optical properties.^10–13^ However, graphene lacks a true phase transition with distinct structural states and large optical contrast, which are essential for nonvolatile THz switching. These limitations have led to increased attention toward alternative PCMs, particularly chalcogenide-based systems, which provide high optical contrast along with stable, nonvolatile operation.^14^

Ge_2_Sb_2_Te_5_ (GST), a representative chalcogenide phase-change compound, has attracted wide interest due to its rapid and reversible solid–solid transition accompanied by pronounced differences in optical constants between the amorphous and crystalline states. These unique properties have established GST as a good candidate material in optical data storage and reconfigurable photonic devices.^15,16^ Until recently, its nonvolatile switching capability and large modulation depth have highlighted its promise for THz device applications.^17–22^

Recent progress in THz metasurface technology has further emphasized the role of GST in sensing-oriented applications. GST-integrated metasurfaces enable dynamic control of resonance characteristics through reversible phase transitions, supporting functionalities such as switching, absorption tuning, and resonance modulation.^23–25^ These capabilities are particularly advantageous for THz sensing, where resonance shifts induced by environmental changes or analyte interactions can be utilized for label-free detection.^26^ Moreover, GST-based absorbers and resonators provide strong field confinement and high optical contrast, leading to enhanced sensitivity and adaptive sensing without requiring structural modification.^27,28^ Recent developments in programmable and reconfigurable metasurfaces further reinforce the potential of GST for multifunctional THz sensing platforms.^29^

Despite these advantages, several challenges remain. Practical limitations of GST include the strong dependence of phase transition behavior on excitation conditions and the relatively high energy required for switching. Achieving high-contrast phase states under rapid heating also remains challenging (see Supporting Information of ref 30). These constraints highlight the need for improved material design to enable efficient, low-energy, and reliable THz device operation.

To address these challenges, numerous doping strategies have been explored to tune the phase-change behavior of GST thin film. Indium (In) and copper (Cu) doping improve thermal stability and refine the microstructure,^31,32^ while silicon (Si) and nickel (Ni) doping enhance energy efficiency and radio frequency compatibility.^33,34^ More recently, tin (Sn) doping has been shown to lower the activation energy for crystallization, resulting in ultrafast switching.^35,36^ Collectively, these studies demonstrate the versatility of doped GST systems in tuning material properties. Nevertheless, most efforts have focused on memory and optical storage, with relatively few works directed toward exploiting doped GST for a THz metasurface and sensing platform.

Recent studies have demonstrated GST-based THz devices such as switchable metasurfaces, resonators, modulators, absorbers, and tunable lenses. Nevertheless, the use of GST as an active medium for tunable THz sensing remains relatively underexplored. Existing approaches to resonance tuning often rely on geometric modifications, such as adjusting resonator dimensions or layer thickness, which limit scalability and adaptability.

In this work, Sn was incorporated into GST thin films to develop a material platform for tunable THz sensors. The phase-change dynamics, behavior, and dielectric response were investigated, and the experimentally obtained optical parameters were implemented in simulations of a split-ring resonator (SRR) metasurface containing a GST layer beneath a gold resonator. The results demonstrate that phase switching in Sn-incorporated GST produces measurable resonance shifts comparable to structural tuning, while requiring lower thermal energy. These findings highlight Sn-incorporated GST as a promising candidate for reconfigurable THz sensing platforms.

## 2 Experimental Details

The GST and Sn-incorporated GST thin films were prepared on alumina ceramic (Al_2_O_3_, 96%; Able Target Limited) using a comagnertron sputtering system (ATC 2000-F, AJA International, Inc.). The deposition chamber was evacuated to a base pressure of 10^–6^ Torr, and the substrates were further treated with argon plasma bombardment for 10 min to remove residual surface contamination. During the film growth, the operating pressure and argon flow rate were maintained at 10 mTorr and 30 sccm, respectively. A pulsed direct current (DC pulse) power supply was applied to the GST target (99.99% purity, 2-in. diameter, 0.250-in. thick) at 50 W, while the Sn target (99.998% purity, 2-in. diameter, 0.250-in. thick) was powered at 0, 12, and 24 W to adjust the incorporating level. All prepared film samples were deposited to a film thickness of approximately 500 nm. After the deposition, the as-deposited GST and Sn-doped GST thin films were annealed on a hot plate at 150 and 250 °C for 15 min to examine the amorphous–crystalline phase transition. Note that the undoped films are denoted as AS-GST (as-deposited), GST-150, and GST-250 for the respective annealing conditions. Similarly, Sn-incorporated films prepared at 12 W are labeled AS-Sn12W-GST, Sn12W-GST-150, and Sn12W-GST-250, while those fabricated at 24 W are referred to as AS-Sn24W-GST, Sn24W-GST-150, and Sn24W-GST-250.

The composition of the prepared films was analyzed using FE-SEM and EDS (HITACHI SU8030). Structural characterization was performed using GIXRD (GIXRD: Rigaku, TTRAX III) patterns. X-ray photoelectron spectroscopy (XPS) measurements were carried out at the SUT-NANOTEC-SLRI joint research facility on beamline 3.1 at the Synchrotron Light Research Institute (Public Organization, Thailand). A scanning XPS microprobe (PHI 5000 VersaProbe II, ULVAC-PHI) was operated using monochromated Al Kα radiation (*hν* = 1486.6 eV). The X-ray beam was focused to a spot size of approximately 100 μm, and photoelectrons were collected using a concentric hemispherical analyzer at a takeoff angle of 45° with respect to the normal surface. Charge neutralization was applied using low-energy electrons and Ar^+^ ions. The binding energy scale was calibrated using the C–C component of the C 1s peak at 284.8 eV. The overall energy resolution was approximately 0.6 eV, and the base pressure in the analysis chamber was maintained at ∼4 × 10^–9^ mbar. Spectral fitting and background subtraction were performed using EX3 ms software (Excel VBA-based).^37^ The optical properties in the THz range are studied using THz-Time Domain Spectroscopy (TDS-Toptica TeraFlash). Finally, the design and electromagnetic response of the tunable THz metasurface incorporating GST as a phase-change material are simulated using the CST Microwave Studio suite.

## 3 Results and Discussion

Figure 1(a) presents the surface morphology of the as-deposited GST and Sn-incorporated GST thin films grown on alumina substrates at different Sn sputtering powers (12–24 W). All samples exhibited relatively rough surfaces. The corresponding EDS spectra of the films prepared at 0, 12, and 24 W are shown in Figure 1(b). Peaks from Al and O, originating from the alumina substrate, were consistently observed in all spectra. In addition, a small C signal was detected, which can be attributed to surface contamination. Figure 1(c) summarizes the atomic composition of the films, excluding contributions from Al, O, and C. The results indicate a clear increase in Sn content with increasing sputtering power, accompanied by variations in the relative concentrations of Ge, Sb, and Te. Additional EDS elemental mapping of the as-deposited GST and Sn-incorporated GST thin films was carried out to examine the spatial distribution of constituent elements, as shown in Figure 1(d). The results show that Ge, Sb, and Te are uniformly distributed in the as-deposited GST film. For the Sn-incorporated samples (12 and 24 W), the elemental maps indicate a homogeneous distribution of Sn across the film surface, without observable segregation. These results confirm that Sn is uniformly incorporated within the GST matrix at both doping levels.

**Figure 1:**
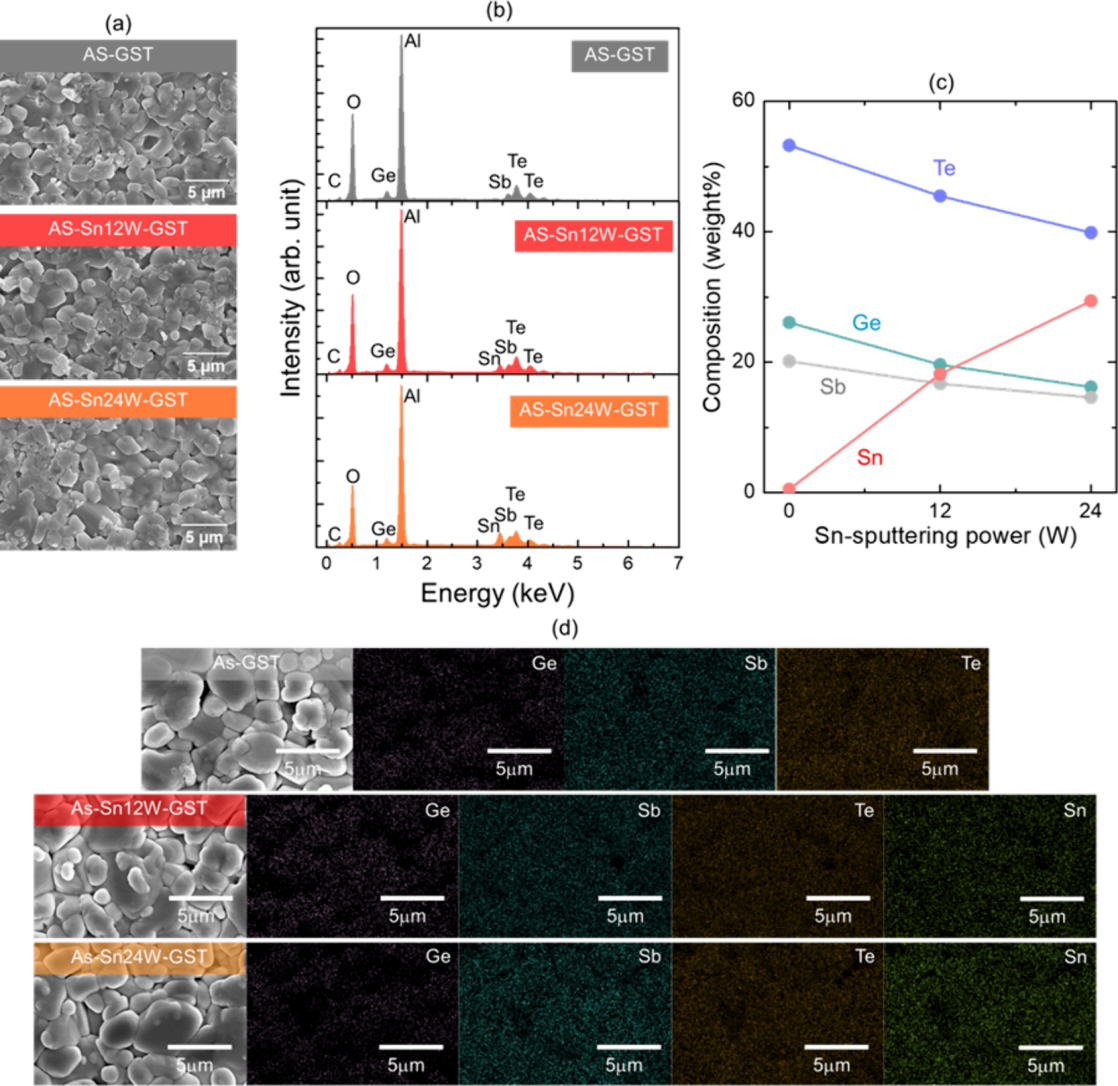
(a) FE-SEM images, (b) the EDS spectra, (c) chemical composition, and (d) EDS elemental mapping of as-deposited GST and Sn-incorporated GST thin film at different sputtering powers.

The phase structure of the films deposited on alumina substrates was characterized by GIXRD, as shown in Figure 2. All as-deposited GST and Sn-incorporated GST thin films exhibited a broad diffuse peak, confirming their amorphous nature. Note that several weak reflections observed 2θ at 25.5°, 35.2°, 37.8°, 52.6°, and 57.5° originated from the alumina substrate (JCPDS No. 42-1468).

**Figure 2:**
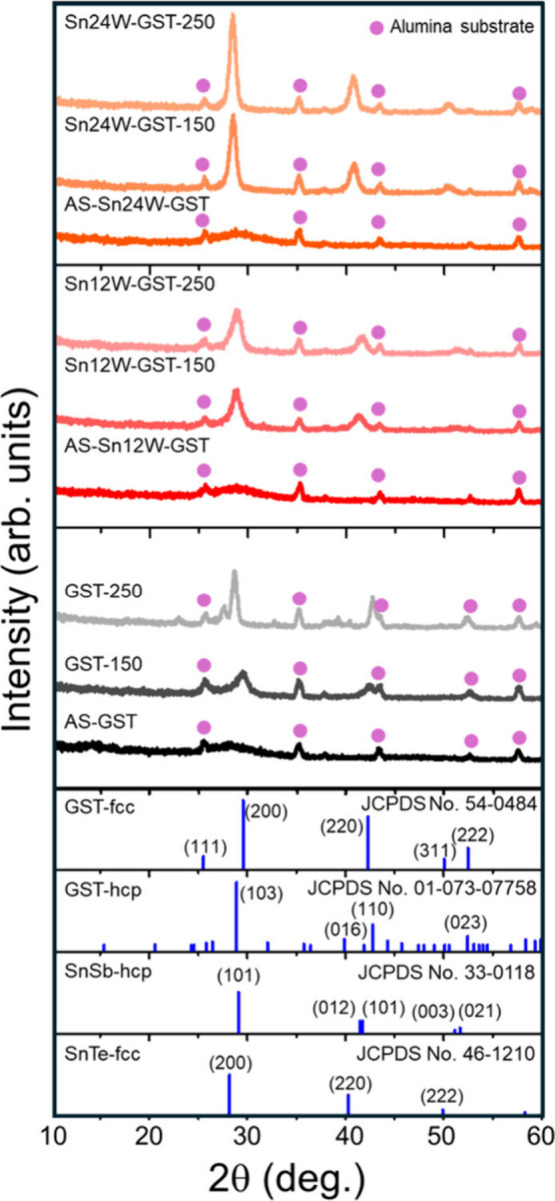
GIXRD patterns of GST and Sn-incorporated GST thin films in the as-deposited and after annealing at 150 and 250 °C.

Upon annealing, the undoped GST film revealed clear structural evolution. At 150 °C (Figure 2(a)), distinct diffraction peaks emerged at 2θ = 29.5° and 42.4°, which correspond to the (200) and (220) planes of a face-centered cubic (FCC) phase (JCPDS No. 54-0484). When the annealing temperature increased to 250 °C, additional peaks appeared at 28.7°, 39.2°, and 42.7°, indexed to the (103), (110), and (023) planes of a hexagonal close-packed (HCP) phase (JCPDS No. 01-073-07758). These observations indicate a progressive transformation from the FCC to the HCP phase with increasing temperature.

Figure 2(b) and (c) presents the GIXRD patterns of Sn-incorporated films prepared at sputtering powers of 12 and 24 W, respectively. After annealing at 150 and 250 °C, the 12 W film displays a more defined yet still broadened peak near 29°, arising from the overlap between the hcp (013) plane of GST and the hcp (101) plane of SnSb. The reflections at 41.8° could be attributed to the mixed crystallinity between the (012) and (110) planes. Additionally, reflection at 51.8° corresponds to the (021) planes of SnSb (JCPDS No. 33-0118). In contrast, the 24 W film exhibits sharper and more intense reflections at 28.2°, 40.8°, and 50.4°, assigned to the (200), (220), and (222) planes of the SnTe crystalline phase (JCPDS No. 46-1210). The slight broadening of the ∼28° peak can be attributed to overlapping contributions from the hcp (103) plane of GST and the fcc (200) plane of SnTe.

To further quantify the structural evolution, the crystallite size was estimated using the Scherrer equation.^38^ For consistency, the analysis was performed using the most prominent diffraction peak of each sample, in order to minimize uncertainties arising from peak overlap and ensure reliable comparison. For the undoped GST film, the crystallite size increases significantly from ∼5.6 nm at 150 °C (FCC phase) to ∼12.3 nm at 250 °C (HCP phase), indicating pronounced grain growth associated with the phase transition.

In comparison, the Sn-incorporated film at 12 W exhibits relatively small crystallite sizes, increasing slightly from ∼6.1 nm to ∼6.3 nm with increasing annealing temperature, suggesting that the presence of mixed phases (GST and SnSb) and peak overlap suppresses grain growth and limits the development of long-range crystalline order.

For the 24 W film, relatively large crystallite sizes are observed, increasing slightly from ∼11.1 nm to ∼11.6 nm upon annealing. This trend is consistent with the enhanced peak intensity and sharper diffraction features observed at higher annealing temperatures, indicating improved crystallinity and the formation of more stable crystalline domains associated with the SnTe phase. The variation in crystallite size obtained from different diffraction planes is attributed to anisotropic crystal growth and possible strain effects, which are commonly observed in GST-based thin films.

Sn incorporation alters the crystallization behavior of GST, modifying its transformation pathway rather than directly improving crystallinity. Diffraction analysis reveals the coexistence of several crystalline phases, mainly SnTe and SnSb, together with residual GST reflections, indicating that Sn atoms tend to bond preferentially with Te and Sb during thermal processing. Such mixed-phase formation suggests that Sn changes the atomic rearrangement dynamics and promotes heterogeneous nucleation within the film. The relatively low bond dissociation energy of the Sn–Te bond (359.8 kJ mol^–1^) compared with the Ge–Te bond (456 kJ mol^–1^) further facilitates bond rupture and atomic diffusion during annealing. Consequently, crystallization begins at a lower temperature, governed by the emergence of Sn-rich phases with weaker bonding strength. Thus, Sn incorporation does not strengthen the crystallinity of GST itself but rather assists in phase development through mixed-phase interactions, thereby reducing the effective crystallization temperature of the composite system.

Figure 3(a–e) shows XPS spectra that were deconvoluted using combinations of Gaussian (G), Gaussian–Lorentzian (GL), and tailed GL peak shapes, after background subtraction using Shirley and Tougaard functions together with polynomial fitting.^39,40^ The C 1s spectrum in Figure 3(a) was fitted with a single Gaussian component on a Shirley-type background and assigned to the C–C peak, which was used as the binding-energy reference. The Sb 3d spectrum in Figure 3(b) overlaps with the O 1s contribution, particularly in the Sb 3d_5/2_ region. To separate these signals, the Sb 3d doublet was fitted under constraints of fixed spin–orbit splitting and relative intensity ratio, based on spin–orbit coupling and multiplicity, respectively. In addition, the O 1s peak was modeled as a single component located at 531.5 eV with a full width at half-maximum (fwhm) of 1.8–2.5 eV. The lower-binding-energy component of each Sb doublet was assigned to Sb(0), including Sb–Sb or Sb-M (M = other metals), whereas the Sb­(III) component was identified as the major chemical state.^41^ The Sn 3d spectra in Figure 3(c) show that Sn(0) appears at the lower-binding-energy side, while Sn­(IV) becomes the dominant chemical state as the Sn target power increases.^42^ The Te 3d spectra in Figure 3(d) contain distinguishable contributions from Te(0) and Te­(IV), although the peak positions vary slightly.^43^ These shifts may be associated with the coexistence of amorphous and crystalline phases in the films.^44^ The Te(0) contribution is minor in the films without Sn and increases with increasing Sn target power, while the Te­(IV) contribution shows the opposite trend. The Ge 3d region in Figure 3(e) overlaps with the Sb 4d signal. To extract the Ge contribution, the Sb 4d doublets were reconstructed using the chemical-state information obtained from the Sb 3d spectra for two species, Sb(0) and Sb­(III), and the Ge 3d peak was modeled without spin–orbit splitting.^45–47^ Annealing causes only slight variations in both elemental composition and chemical-state distribution. Figure 3(f) shows that carbon and oxygen dominate the surface composition for all films, which is consistent with surface exposure to air during transfer to the XPS chamber, noting that XPS is sensitive to a depth of about 3 nm.^48^ The metallic elements are present in the range of approximately 5–25 atom %, except for Sn, whose content increases from 0 to about 25 at. % with increasing Sn target power. The compositional trends of the metallic elements as a function of Sn content are consistent with the EDX results obtained from the as-deposited films discussed above, despite surface modification caused by carbon and oxygen adsorption. These surface-sensitive XPS results can be further correlated with the bulk structural information obtained from GIXRD. The GIXRD patterns indicate a progressive increase in the SnTe phase with increasing Sn content, consistent with the observed increases in the Te(0) and Sb(0) components in the XPS spectra, accompanied by a reduction in their oxidized states. The presence of oxidized species in XPS is mainly attributed to surface exposure to ambient conditions or high vacuum deposition, which can partially mask the underlying chemical bonding states. In contrast, GIXRD probes the film’s bulk and is therefore more sensitive to crystalline phase formation. Notably, the increase in the Te(0) contribution is more pronounced than that of Sb(0), which supports the dominant formation of Sn–Te bonding and is consistent with the increasing SnTe phase observed in GIXRD. Meanwhile, the relative decrease in Ge(0) with increasing Sn content suggests a modification of the local bonding environment involving Ge–Te units, rather than a direct phase substitution. This difference highlights the complementary nature of XPS and XRD, where surface chemical states and bulk crystalline phases may not be fully identical due to the limited probing depth of XPS and the influence of surface oxidation.

**Figure 3:**
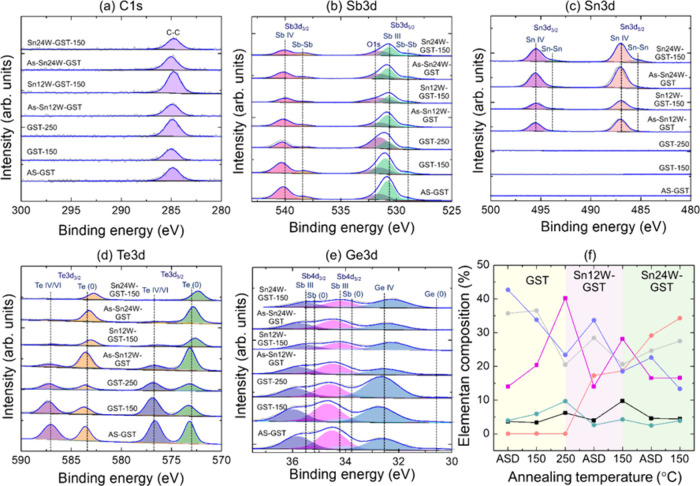
XPS spectra of undoped and Sn-incorporated GST thin films: (a) C 1s, (b) Sb 3d with overlapping O 1s, (c) Sn 3d, (d) Te 3d, and (e) Ge 3d; (f) corresponding elemental composition as a function of annealing temperature.

The THz optical properties of the GST and Sn-incorporated GST thin films are characterized using THz-TDS. This noncontact technique enables the broadband measurement of THz transmission through thin films, making it well-suited for analyzing phase-change materials in both their amorphous and crystalline states. Figure 4(a) shows the time-domain THz signal of the GST and Sn-incorporated GST thin films under various annealing conditions. In each graph, two distinct features are observed: a primary peak corresponding to direct THz transmission, and a secondary peak arising from internal reflection within the thin film and the substrate. For the GST samples, the primary peak intensity decreases progressively with increasing annealing temperature, from room temperature to 250 °C. The secondary peak, related to internal reflection, remains visible up to 150 °C but disappears completely at 250 °C. The vanishing of the internal reflection peak is attributed to enhanced absorption and, or a refractive index mismatch, consistent with the formation of a more metallic crystalline phase at high temperatures. Notably, the THz response at room temperature and 150 °C exhibits only minor change, suggesting that the crystallization threshold of pure GST is not significantly exceeded at 150 °C.

**Figure 4:**
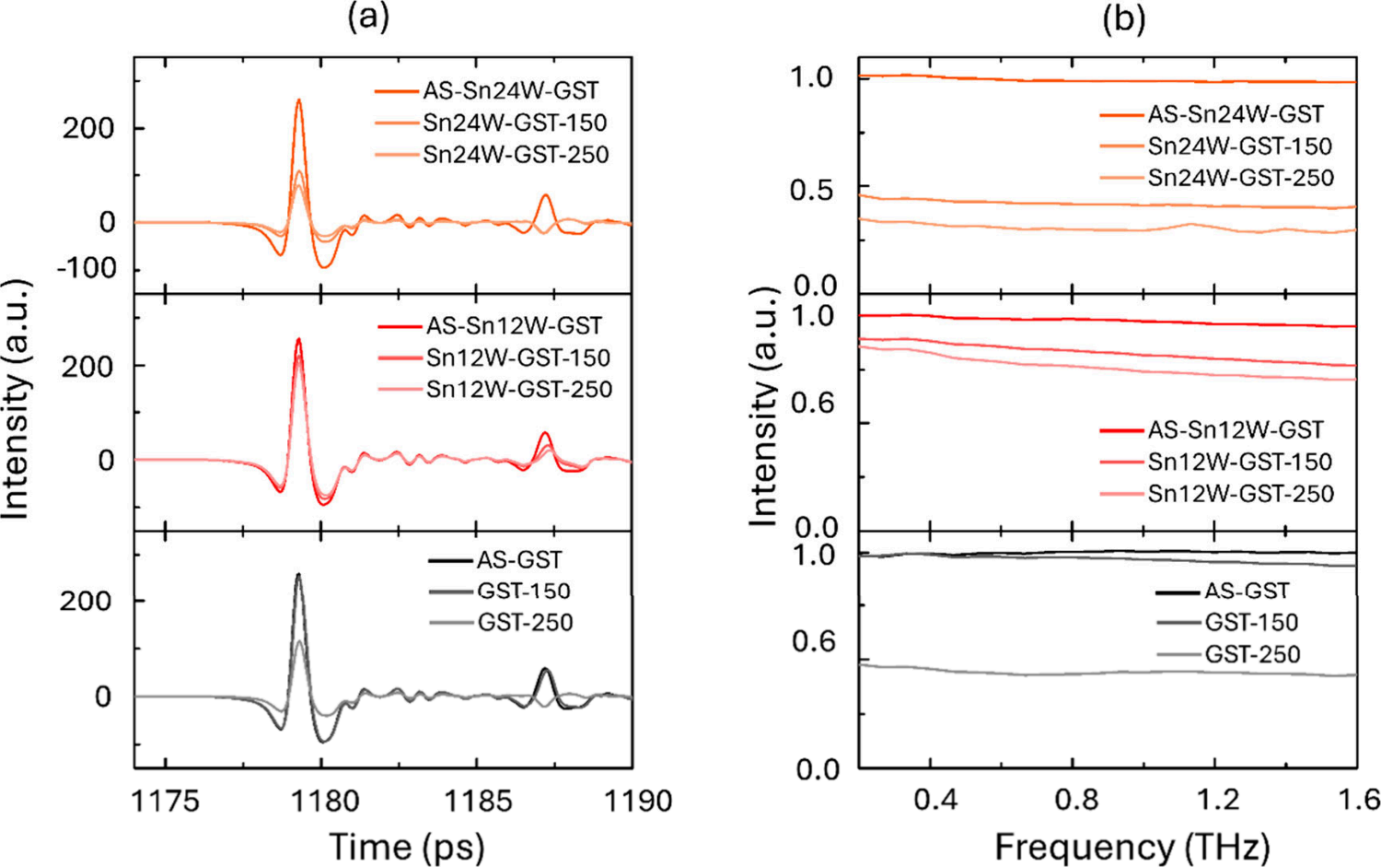
(a) Time-domain waveforms and (b) corresponding transmission spectra of GST thin films in pure GST and Sn-incorporated GST thin films, measured by THz-TDS.

The Sn-incorporated GST films deposited at 12 W–Sn sputtering power (middle panel of Figure 4(a)) exhibit an accelerated crystallization process. At 150 °C, both the primary and secondary peak intensities are substantially reduced compared to the undoped films, indicating that Sn doping facilitates partial crystallization at a lower temperature than in the undoped case. At 250 °C, the THz signal becomes even more pronounced, consistent with the evolution toward a strongly absorbing crystalline state.

A more pronounced effect is observed in Sn-incorporated GST films deposited at 24 W–Sn sputtering power (top panel of Figure 4(a)). At 150 °C, the primary peak intensity is significantly reduced, and the internal reflection peak completely vanishes. This behavior indicates stronger interaction with incident THz radiation, consistent with enhanced free-carrier density and absorption in the crystalline phase. These observations align with XPS and GIXRD results, which demonstrate that Sn doping promotes crystallization and metallic bonding at lower annealing temperatures.

To further interpret the observed dynamics, a Fourier transformation was applied to the time-domain data, extracting the transmission spectra shown in Figure 3(b). For the undoped samples (bottom panel), a clear contrast is observed between the amorphous state (room temperature), which shows high THz transmission, and the crystalline phase (250 °C), which exhibits strong attenuation. The intermediate 150 °C condition exhibits a transmission spectrum closely resembling that of the amorphous phase, further supporting the conclusion that crystallization is incomplete at this temperature in the absence of Sn.

In contrast, the Sn-incorporated GST thin films demonstrate markedly different behavior. For the 24 W sample (top panel of Figure 4(b)), the film annealed at 150 °C already shows a pronounced transmission decrease from 100% to ∼70%, compared at room temperature and ∼75% for the crystalline pure GST film annealed at 250 °C. This indicates that a substantial phase transformation, accompanied by improved crystalline structure, occurs in the Sn-incorporated film at a significantly reduced thermal threshold. In comparison, the 12 W Sn-incorporated GST films (middle panel) exhibit a more gradual decrease in transmission with increasing temperature, reflecting a weaker but still evident influence of Sn doping. This systematic trend highlights the dose-dependent effect of Sn incorporation in lowering the crystallization temperature and enhancing the THz optical contrast.

To quantitatively analyze the terahertz (THz) response of the GST thin films with a thickness of 500 nm on the Al_2_O_3_ substrate with thickness of 650 μm. Temporal windowing (time-gating) was applied to the primary THz pulses to eliminate subsequent Fabry-Pérot reflections within the thick substrate. After performing a Fast Fourier Transform (FFT) on the gated signals, the complex transmission coefficient is defined as $\mathrm{t̃}\left(\omega \right)=\frac{E_{sample}\left(\omega \right)}{E_{ref}\left(\omega \right)}=A\left(\omega \right)e^{i\Delta \phi \left(\omega \right)}$

The phase shift Δϕ­(ω) between the sample and reference introduced by the GST film can be related to the real part of the refractive index *n*(ω) as

1 $$\Delta \phi \left(\omega \right)=\frac{\omega d}{c}\left[n\left(\omega \right)-1\right]$$

2 $$n\left(\omega \right)=\frac{c\Delta \phi \left(\omega \right)}{\omega d}+1$$

where ω is the angular frequency, *c* is the speed of light in vacuum, and *d* is the thickness of the GST film. Given that the film thickness is much smaller than the THz wavelength, we employed Tinkham’s approximation^49^ to extract the complex optical conductivity *σ̃*(ω) = σ_1_ + *iσ*_2_. This model effectively accounts for the Fresnel reflections at the interfaces of the thin film on a substrate and the optical conductivity is given by

3 $$\mathrm{t̃}\left(\omega \right)=\frac{1+n_{s}}{1+n_{s}+Z_{0}\mathrm{\sigma ̃}\left(\omega \right)d}$$

From eq 3, the real part of the optical conductivity σ_1_(ω) can be extracted as

4 $$\sigma_{1}\left(\omega \right)=Re\left[\frac{n_{s}+1}{Z_{0}d}\right]\left(\frac{1}{\mathrm{t̃}\left(\omega \right)}-1\right)$$

where *n*_*s*_ is the refractive index of the substrate and *Z*_*0*_ ≈ 377Ω is the impedance of free space. The extinction coefficient *k*(ω) is then derived from the relationship between the real conductivity and the imaginary part of the permittivity *ε*″:

5 $$\varepsilon^{\prime \prime }=\frac{\sigma_{1}\left(\omega \right)}{\omega \varepsilon_{0}}=2n\left(\omega \right)k\left(\omega \right)$$

6 $$k\left(\omega \right)=\frac{\sigma_{1}\left(\omega \right)}{2n\left(\omega \right)\omega \varepsilon_{0}}$$

Once *n* and *k* are determined, the real permittivity *ε’* of the GST films are calculated as

7 $$\varepsilon^{\prime }=n{\left(\omega \right)}^{2}-k{\left(\omega \right)}^{2}$$

The dielectric parameters of GST and Sn-incorporated GST thin films (12 and 24 W) at room temperature, 150 °C, and 250 °C were extracted from THz-TDS measurements and are summarized at 1 THz in Table 1. As-deposited films exhibit low extinction coefficient, conductivity, and dielectric loss, indicating insulating behavior. With increasing annealing temperature, both GST and Sn-doped GST films show a pronounced increase in conductivity and imaginary permittivity, reflecting enhanced free-carrier response associated with partial crystallization.

**Table 1:** Dielectric Properties of GST Thin Films at 1 THz Extracted from THz-TDS Measurements

sample	*n*	*k*	σ	*ε*′	*ε*″
AS-GST	10.01	0.00	0.00	100.21	0.00
GST-150	20.29	0.15	345.68	411.79	6.21
GST-250	20.30	4.13	10577.24	395.04	167.76
AS-Sn12W-GST	12.85	0.20	288.36	165.09	5.18
Sn12W-GST-150	18.07	1.12	2250.41	325.11	40.45
Sn12W-GST-250	18.43	1.67	3416.57	336.90	61.41
AS-Sn24W-GST	17.53	0.07	131.08	307.43	2.36
Sn24W-GST-150	26.14	4.09	11892.95	666.70	213.78
Sn24W-GST-250	49.43	3.26	17928.63	2432.49	322.28

This significant increase in conductivity and dielectric loss can be attributed to the structural transition from an amorphous to a crystalline phase, which leads to a higher carrier concentration and improved carrier mobility. In the amorphous phase, charge transport is limited by localized states and structural disorder. In contrast, the crystalline phase exhibits a more ordered lattice, enabling delocalized electronic states and enhanced electrical conductivity. Furthermore, the crystalline GST phase is known to exhibit a metal-like electronic structure, characterized by high carrier density and Drude-like free-carrier behavior in the THz frequency range. This results in increased absorption and dielectric loss, as reflected in the higher imaginary permittivity values. These changes are consistent with the observed evolution of THz response and confirm the strong correlation between phase transition and dielectric properties in GST-based films.

Sn doping significantly modifies the terahertz response, with Sn24W-GST exhibiting the highest refractive index, conductivity, and dielectric loss at 250 °C, highlighting the strong tunability of dielectric properties through compositional engineering and thermal treatment. These extracted dielectric parameters were subsequently implemented in CST Microwave Studio simulations to investigate the thermally tunable resonance behavior of the split-ring resonator (SRR) metasurfaces.

In this part, we integrate the phase-change GST investigated in a previous session with a terahertz (THz) split-ring resonator (SRRs). SRRs have been extensively employed in terahertz (THz) and optical devices due to their strong localized resonances, which enable efficient control of electromagnetic wave propagation at subwavelength scales. In particular, SRRs serve as fundamental building blocks for filters and resonators, providing sharp and tunable frequency responses, as well as for highly sensitive sensors, where the resonance shifts in response to changes in the dielectric environment. These unique properties make SRRs especially suitable for integration with active materials. Here, we employ SRR-based metasurfaces to achieve tunable resonance frequency behavior, demonstrating the feasibility of dynamically reconfigurable THz and optical components that can function as switchable filters, resonators, and sensors.

Generally, the resonance frequency of a split-ring resonator (SRR) can be tuned by modifying its geometric parameters, such as the unit cell periodicity (p) of 50 μm, side length (a) of 40 μm, width of the square ring of 10 μm, and the split gap (g), as illustrated in Figure 5(a). In this structure, a 500 nm-thick GST film is deposited on top of the substrate, and the gold SRR is patterned directly on the GST layer. The SRR has a side length (a) of 40 μm with a ring width of 10 μm, and a split gap (g) that determines the capacitive coupling in the resonator. Simulations using CST Microwave Studio show that the resonance frequency can be shifted by approximately 0.1 THz when the gap (g) is increased from 3 to 9 μm, as presented in Figure 5(b). However, this structural tuning requires the fabrication of new sensors with different geometries for each desired frequency shift.

**Figure 5:**
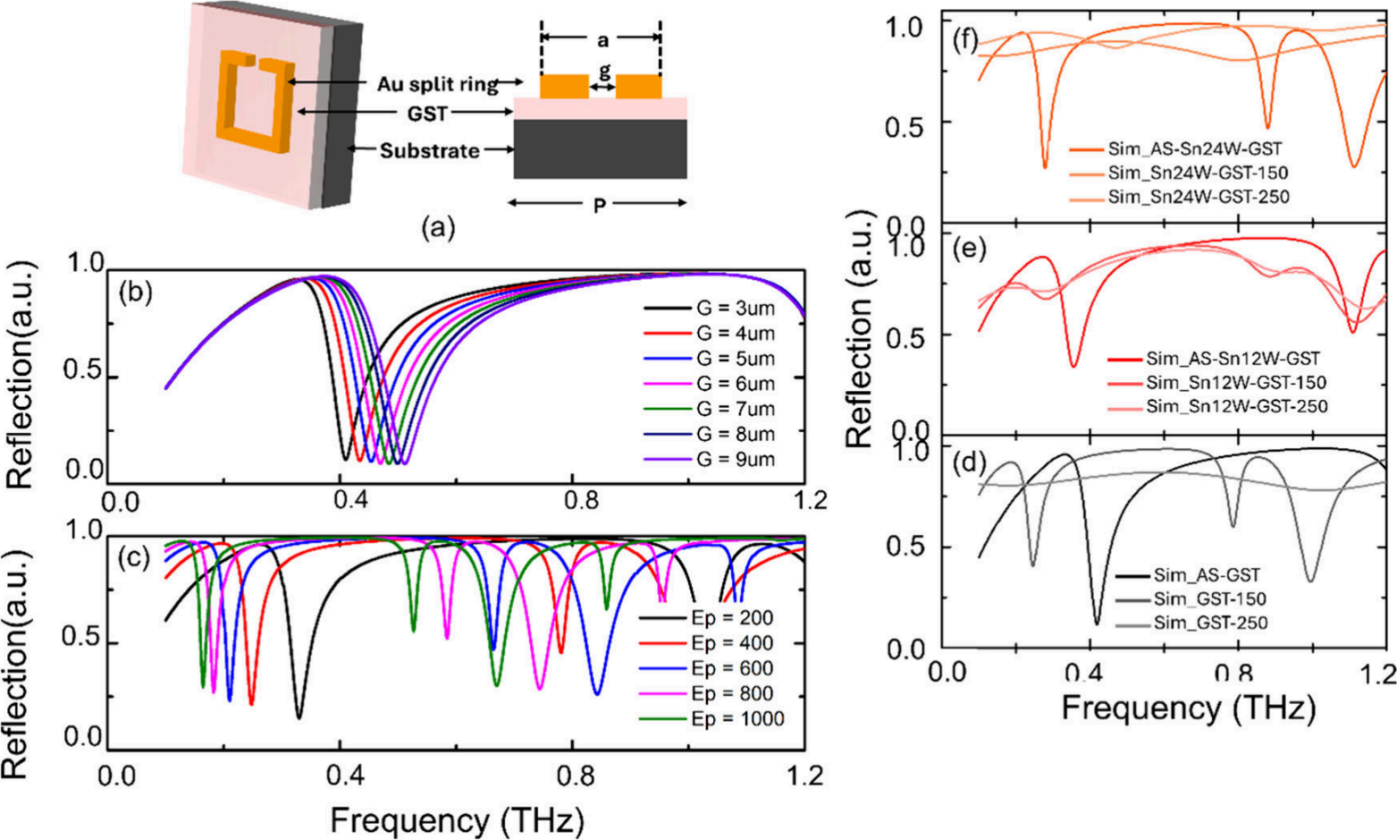
(a) Schematic illustration of the GST integrated split-ring resonator (SRR) unit. Simulated reflection spectra with (b) varying gap widths and (c) different real permittivity value (ε′). Simulated responses obtained using experimentally derived dielectric parameters of (d) pure GST, (e) Sn-incorporated GST (12 W), and (f) Sn-incorporated GST (24 W) extracted from THz-TDS measurements.

To illustrate the concept of dynamic tunability, we first performed simulations of an SRR metasurface integrated with the phase-change material GST, demonstrating that the resonance frequency can be tuned without the need to fabricate new sensor structures. As depicted in Figure 5(a), a thin GST film was inserted between the substrate and the Au split ring while keeping the split gap fixed at 3 μm. By varying the real permittivity (ε′) of GST from 200 to 1000 with a loss tangent of zero, the resonance frequency could be adjusted within a range of 0.16 THz, as shown in Figure 5(c). This simulation demonstrates the feasibility of tuning the SRR resonance via permittivity modulation.

Furthermore, experimentally extracted dielectric parameters of GST and Sn-incorporated GST thin films, obtained from THz-TDS measurements, were incorporated into CST Microwave Studio simulations. The complex refractive index and conductivity values were directly used to represent the realistic material response at different phase states. Using these parameters, the resonance behavior of the GST-based SRR metasurfaces was systematically investigated, as shown in Figure 5(d)–(f).

Annealing at 150 °C induces a distinct shift of the first-order resonance frequency by approximately 0.16 THz, comparable to the tuning range achieved through structural gap modification (Figure 5(c)). Although the resonance dip becomes shallower and additional features appear, the primary resonance remains identifiable, demonstrating that temperature provides an effective tuning mechanism. This thermally induced resonance shift is particularly beneficial for sensing applications, where frequency modulation is the key performance metric. However, the resonant nature of this mode will be further validated by electric-field distributions presented in the subsequent section. Upon further annealing to 250 °C, the resonance features are strongly suppressed and eventually disappear. This suppression is attributed to the pronounced increase in electrical conductivity associated with the crystalline phase, leading to metallic-like behavior that significantly damps the resonant response of the SRR structure.

Sn incorporation markedly alters the resonance characteristics, as shown in Figure 5(e) and (f). In the as-deposited state, Sn-incorporated GST films (12 and 24 W) exhibit higher refractive index values, leading to a downward shift of the first-order resonance frequency compared to pristine GST. Notably, resonance suppression occurs at lower annealing temperatures (≈150 °C), indicating that Sn doping accelerates the transition toward a highly conductive, metallic electromagnetic response. This behavior enables a clear on–off switching of the SRR resonance with reduced thermal stimulus, highlighting the potential of Sn-incorporated GST for low-energy, thermally controlled terahertz switching and reconfigurable metasurface devices. The reflection spectra of the proposed split-ring resonator (SRR) metasurface shown in Figure 5(d) and (f) are further investigated to analyze electromagnetic behavior. A Normally incident plane wave with its electric field polarized parallel to the SRR gap is used to confirm strong capacitive coupling across this gap. A tetrahedral mesh with local refinement around the SRR gaps and edges is applied to accurately capture the strong field variations in these regions. The simulated electric field distributions at the frequencies are presented in Figure 6. For the undoped GST film annealed at room temperature, strong electric field confinement and enhancement are observed across the SRR split gaps at the resonance frequency near 0.4 THz, as illustrated in Figure 6(a). This confirms that the incident wave excites an electric dipole resonance, producing pronounced localized capacitive effects at this frequency. In contrast, no significant field confinement is observed at 0.25 THz under the same condition. However, when the undoped GST film is annealed at 150 °C, the resonance behavior shifts, with a localized electric dipole response appearing at 0.25 THz, while the confinement at 0.4 THz disappears. This shift verifies the phase-change nature of GST and demonstrates its suitability for resonance tunability in SRR-based metasurfaces.

**Figure 6:**
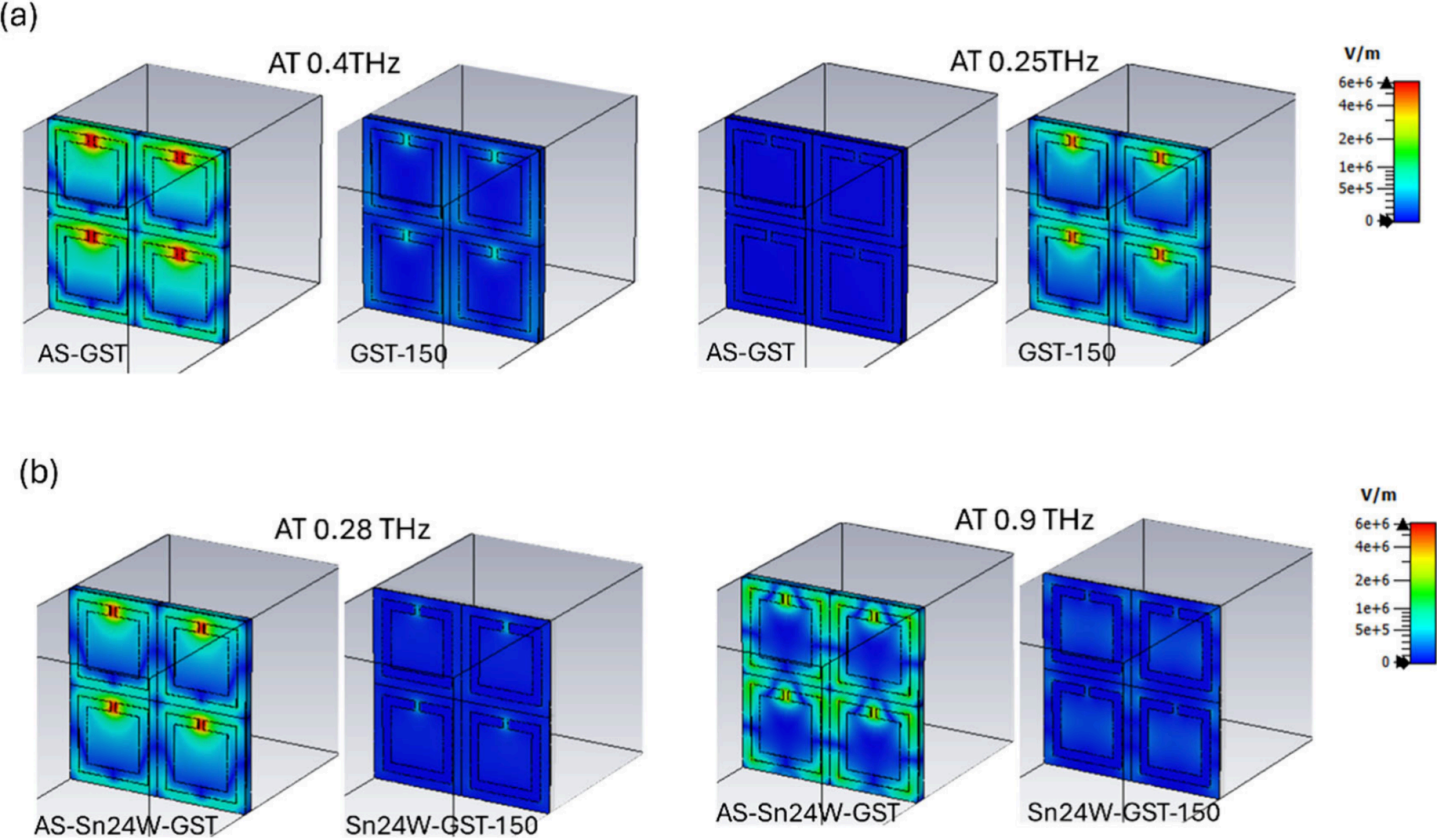
(a) The electric field distributions of the undoped GST films, and (b) Sn-incorporated GST films (24 W) SRR metasurface at room and 150 °C annealing temperature.

Figure 6(b) shows the electric field distributions of SRRs incorporating Sn-doped GST films (deposited at 24 W) at both room temperature and after annealing at 150 °C. At room temperature, strong electric field localization is observed in the resonator gap at the resonance frequency of 0.28 THz, similar to the undoped GST case. Additionally, field confinement is still present at 0.9 THz, although weaker, which can be attributed to higher-order resonant absorption in the SRR structure.

In contrast, the Sn-doped GST film annealed at 150 °C exhibits negligible electric field confinement at both 0.28 THz and 0.9 THz. This behavior is due to the increased electrical conductivity and metallic-like characteristics of the material after annealing, which enhance reflection at the SRR interface and suppress resonance formation. These results demonstrate the improved on–off switching performance of Sn-doped GST compared to undoped GST film, indicating its strong potential for electromagnetic switching applications.

Integrating GST with SRR metasurfaces enables dynamically tunable THz sensors with enhanced sensitivity and adaptability. This approach opens the possibility of creating spectral fingerprints of samples, particularly when combined with machine learning algorithms. Phase-change materials, such as GST, thus offer a promising route toward next-generation, reconfigurable THz sensing platforms for applications in spectroscopy, chemical and biological detection, and security screening.

## 4 Conclusions

This work examined GST and Sn-incorporated GST thin films deposited on alumina substrate to understand the influence of Sn on structure and THz performance for tunable THz sensing. FE-SEM revealed rough surface roughness, while EDS confirmed that Sn content increased with sputtering power. The GIXRD result showed that Sn modifies the crystalline route of GST, forming mixed SnTe and SnSb phases together with residual GST, indicating preferential bonding between Sn, Te, and Sb that enables phase formation at lower temperatures through weak Sn–Te bonds. XPS spectra further supported this trend, showing a downward shift in the Te 3d peaks, suggesting an evolution toward increased metallic character and modified local bonding environments involving Sn–Te interactions. It should be noted that XPS primarily probes surface chemical states, while GIXRD reflects bulk structural phases; thus, the combined results provide complementary insight into Sn-induced phase evolution and bonding behavior. THz-TDS analysis demonstrated strong dielectric contrast between amorphous and crystalline states, with Sn incorporation amplifying the tunability. Simulations of SRR metasurfaces using measured dielectric data showed a resonance shift of about 0.16 THz from phase transition GST thin films. Additionally, Sn-incorporated GST could improve modulation efficiency with reduced thermal input. Overall, Sn incorporation provides a practical route to control phase evolution and resonance behavior in GST-based materials, contributing to advancing tunable THz photonics devices.
